# A unique pediatric thoracic fibrosarcoma: Case report and successful therapeutic strategy

**DOI:** 10.1016/j.ijscr.2025.111999

**Published:** 2025-10-02

**Authors:** Khaled Alomar, Kamar Shaker, Nidal Alkhani

**Affiliations:** a Pediatric and Neonatal Surgery, Syria; b Maternity and Children's Hospital, Hama, Syria; c Damascus University, University pediatrics Hospital, Damascus, Syria; d Head of NICU, Maternity and Children's Hospital, Hama, Syria

**Keywords:** CaseReport-pediatric-fibrosarcoma, Congenital-infantile

## Abstract

**Introduction and significance:**

Congenital Infantile Fibrosarcoma (CIFS) is an exceptionally rare pediatric malignancy, representing approximately 10 % of all soft tissue cancers in young children. Its clinical manifestations vary according to tumor location. When tumors occur in unusual anatomical sites, symptoms may be misleading and delay proper diagnosis and treatment.

**Case presentation:**

We report an 11-month-old male with a month-long persistent dry cough and high fever unresponsive to antibiotics. Chest CT revealed a pleural mass with mild effusion. Thoracotomy achieved complete excision (R0). Histopathology showed low-grade fibrosarcoma; immunohistochemistry was positive for Vimentin and SMA, focally CD34, and negative for S100, Desmin, and Myogenin, with low Ki-67 (<3 %). ETV6-NTRK3 fusion testing was unavailable. The patient received six cycles of adjuvant VAC chemotherapy and tolerated treatment well, with only mild transient neutropenia.

**Clinical discussion:**

Diagnosis depends on histopathological and immunohistochemical analysis, as CIFS mimics several other soft tissue sarcomas. Our review of medical literature found no prior cases of CIFS originating in the pleura, underlining the uniqueness of this case. Management followed a standard multidisciplinary approach consisting of complete surgical excision and adjuvant chemotherapy (VAC protocol). At 12-month follow-up, the patient remained recurrence-free.

**Conclusion:**

Persistent pneumonia-like symptoms unresponsive to standard treatment in infants should prompt consideration of underlying malignancy. Early recognition and prompt management are essential for improving outcomes in such rare presentations.

## Highlights

• Congenital infantile fibrosarcoma (CIFS) is a rare tumor presenting shortly after birth and developing in early childhood.
• Pleural localization of CIFS is exceptionally uncommon, with only few cases described in the literature.
• Persistent pneumonia-like symptoms not responding to antibiotics should prompt consideration of malignant etiologies.
• Surgical removal is the primary treatment, often combined with chemotherapy.
• Local recurrence occurs in up to 40 % of cases.

## 1 Introduction

Solid tumors in neonates and newborns are uncommon and predominantly benign. Malignant neoplasms in this population account for merely 2 % of all pediatric cancers. Among these, soft tissue sarcomas—originating from mesenchymal precursors—have the potential to differentiate into muscle, fat, fibrous, or other connective tissues. Fibrosarcomas in infants fall into two distinct categories: Congenital Infantile Fibrosarcoma (CIFS) and the adult-type variant. While they share some histological features, their clinical behavior and outcomes differ significantly( [[1], [2]]).

CIFS is rare, comprising only about 10 % of malignant tumors in children ( [[3], [4]]). It predominantly arises in the extremities and, to a lesser extent, the trunk, head, or neck. Involvement of the chest cavity is exceptionally rare, with no previously documented cases found in the literature.

Accurate diagnosis relies on a multidisciplinary approach that includes clinical evaluation, radiological imaging, and histopathological confirmation. Although adult-type fibrosarcoma tends to appear in older children, typically between 10 and 15 years of age, both forms exhibit spindle-shaped fibroblasts histologically. A distinctive genetic hallmark of CIFS is the presence of a recurrent chromosomal translocation t(12;15), resulting in the ETV6-NTRK3 fusion gene, which is not seen in adult-type fibrosarcoma. This genetic alteration is believed to correlate with a more favorable prognosis ( [5]).

This case is described in accordance with the criteria of SCARE ( [6]).

## 2 Case presentation

### 2.1 Patient information

We report the case of a 11-month-old male infant, born to non-consanguineous parents via elective cesarean section at full term, with normal perinatal history and appropriate developmental milestones. He had no prior chronic illnesses or congenital anomalies. The child was up to date with immunizations and had no significant family history of malignancy. He presented to the Emergency Department with high-grade fever, non-productive cough, tachypnea, and progressively worsening dyspnea. The initial symptoms began 4 months prior to admission, marked by episodes of fever and persistent cough. At that time, he was evaluated at a rural healthcare facility and diagnosed with acute pneumonia, for which he received 12 days of intravenous (IV) antibiotics during hospitalization.

Following initial symptom resolution, the patient experienced a relapse 7 days post-discharge, necessitating readmission and a 21-day course of antibiotics for presumed bilateral pneumonia, which yielded only minimal clinical improvement. Due to the lack of substantial recovery, the patient was referred to our tertiary university hospital for advanced diagnostic workup.

At the time of admission, the infant was found to be tachycardic, tachypneic, and mildly dehydrated. There were no associated gastrointestinal or genitourinary symptoms. The patient's family, drug, and allergy histories were non-contributory.

### 2.2 Clinical findings

Physical examination confirmed tachycardia, tachypnea, prolonged expiratory phase, and bilateral soft rales upon auscultation. Notably, dullness to percussion was observed in the left lower chest, suggestive of localized pathology.

Initial laboratory results were as follows:

Hemoglobin: 9.1 mg/dL.

Mean Corpuscular Volume (MCV): 56 fl.

White Blood Cell Count (WBC): 11.7/μL.

C-Reactive Protein (CRP): 44 mg/L.
Other laboratory parameters were within normal ranges.

## 3 Diagnostic assessment

Chest radiography demonstrated bilateral pulmonary infiltrates, hyperinflation of the left lung, atelectasis of the left middle lobe, and right-sided pleural effusion.

**Fig. 1:**
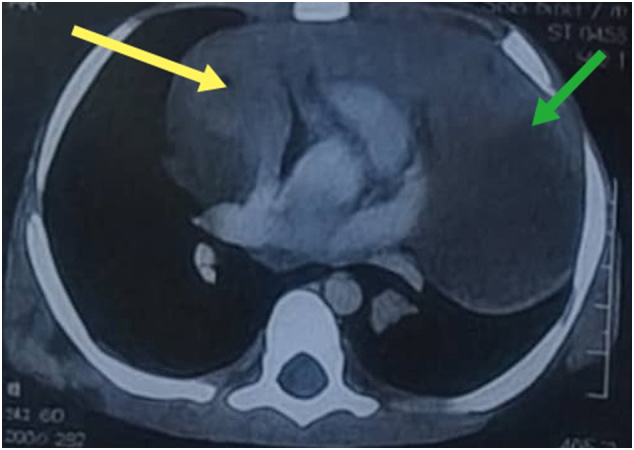
Axial chest computed tomography (CT) scan demonstrating a left-sided thoracic mass (green arrow) associated with mediastinal shift toward the right side (yellow arrow). (For interpretation of the references to colour in this figure legend, the reader is referred to the web version of this article.)

A chest CT scan (Fig. 1) revealed a heterogeneous mass in the posterior region of the left lower lobe, measuring 6 × 67 × 34 mm, causing compression of adjacent lung tissue.

Initial management included:

IV fluid resuscitation.
Broad-spectrum antibiotics.
Full laboratory panel.
Blood grouping and cross-matching for transfusion.
Tumor marker testing showed:
Alpha-fetoprotein (AFP): 53 ng/mL (Normal >10 ng/mL).
Neuron-specific enolase (NSE): 45.17 ng/mL (Normal >17 ng/mL).

To rule out distant metastases, a multi-slice CT (MSCT) of the head, chest, abdomen, and limbs was performed. Results showed no evidence of metastatic spread. These findings were reviewed in a multidisciplinary team (MDT) meeting, where the absence of distant disease was confirmed.

There were no identified risk factors—no maternal exposure to radiation or carcinogens during pregnancy, and no family history of malignancy was reported.

## 4 Therapeutic intervention

Preoperative chemotherapy was considered but ultimately omitted, as the tumor appeared resectable with clear margins on imaging and there was no evidence of metastatic spread. This approach minimized exposure to systemic chemotherapy while prioritizing complete surgical excision (R0), which remains the strongest prognostic factor for survival in CIFS.

**Fig. 2:**
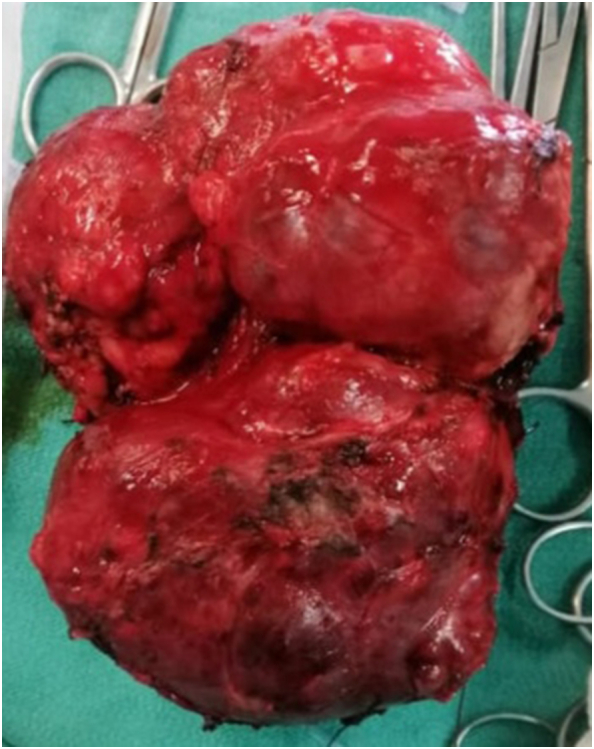
Intraoperative image showing the completely resected tumor**.**

The procedure was performed under general anesthesia without complications. Intraoperatively, a solid pleural mass measuring 9 × 6 × 2 cm was identified in the posterolateral aspect of the left lower thoracic cavity (Fig. 2).

Complete resection was achieved with negative margins (R0). Estimated blood loss was 80 mL. A single chest drain was placed intraoperatively.

The postoperative course included standard perioperative management with IV fluids, analgesia, and chest physiotherapy.

**Fig. 3:**
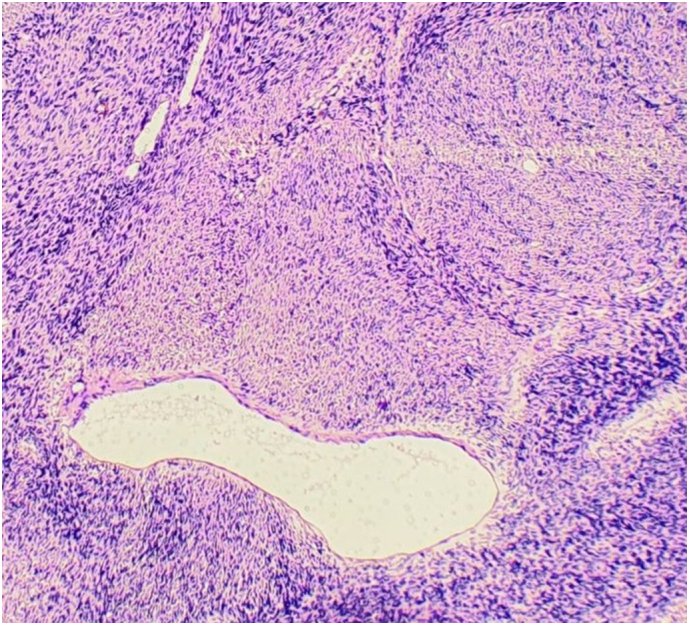
Hematoxylin and eosin (H&E) staining revealing a spindle cell neoplasm consistent with low-grade fibrosarcoma.

Histopathological examination revealed a spindle-cell neoplasm consistent with low-grade fibrosarcoma, along with mild nonspecific pleuritis (Fig. 3). All resected lymph nodes were negative for tumor involvement.

**Fig. 4:**
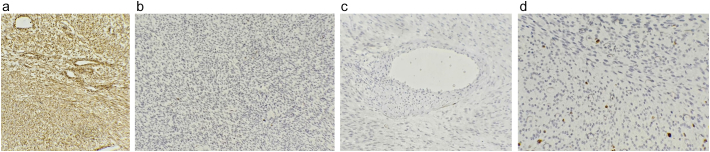
Immunohistochemical analysis: A: Diffuse positivity for vimentin. B: Negative staining for S100 protein. C: Negative staining for desmin. D: Ki-67 proliferation index exceeding 3 % of tumor cells.
Additional staining showed SMA positivity and focal CD34 reactivity, while Myogenin was negative. Molecular confirmation of the ETV6-NTRK3 gene fusion was not performed due to limited resources.

Immunohistochemical analysis supported the diagnosis with the following markers:

Vimentin (VIM): Positive.
S100: Negative.
Desmin: Negative.
Ki-67: <3 % (Fig. 4 D, showing low proliferative index consistent with low-grade fibrosarcoma).

This limitation is clinically relevant, as NTRK inhibitors could have provided a targeted therapy option.

### 4.1 Follow-up and outcomes

Postoperative recovery was uneventful, and the patient was discharged 8 days after surgery. He has been closely monitored as an outpatient for 12 months, during which he was referred to a tertiary oncology center.

He received 6 cycles of adjuvant VAC chemotherapy. The patient tolerated therapy well, with only mild neutropenia and no major adverse effects.

At 12-month follow-up, the patient remained recurrence-free, with no evidence of metastasis on imaging.

However, given that local recurrence occurs in approximately 20–40 % of cases and metastasis may develop in up to 10 % of infants, continued long-term surveillance with periodic imaging and clinical evaluation is strongly recommended.

Clinical timeline of the patient (Table 1).

**Table 1:** Clinical timeline of the patient.

Time Point	Event	Management / Outcome
7 months of age	First episode of fever, cough	Diagnosed with pneumonia, 12 days IV antibiotics
8 months of age	Relapse after 1 week	Readmission, 21 days antibiotics, poor response
11 months of age	Referral to tertiary hospital	CT showed pleural mass
Same admission	Multidisciplinary meeting	Decision for surgical resection
Surgery (11 months)	Thoracotomy, R0 resection	Uneventful recovery
Post-op	Histopathology: low-grade CIFS	IHC: Vimentin+, SMA+, CD34 focal+, Ki-67 < 3 %
1–6 months post-op	Adjuvant VAC chemotherapy (6 cycles)	Mild neutropenia only
12 months follow-up	Imaging and clinical review	No recurrence, no metastasis

## 5 Discussion

### 5.1 Congenital infantile fibrosarcoma (CIFS): A rare pediatric neoplasm

Congenital Infantile Fibrosarcoma (CIFS) is an uncommon soft tissue tumor that typically presents shortly after birth and progresses during early childhood. Representing approximately 1 % of all pediatric tumors and accounting for 5–10 % of soft tissue tumors in infants under one year old, CIFS has an estimated incidence of about 5 cases per million infants. Notably, around one-third of cases are diagnosed in the perinatal period, with nearly 50 % of tumors manifesting before 3 months of age. A secondary incidence peak is observed between the ages of 10 and 15 years ( [[7], [8], [9]]).

Prenatal detection is also possible through imaging modalities such as ultrasound or MRI.

The condition is more prevalent in males, with a male-to-female ratio estimated at 3–4:1, which is consistent with previous reports.

Over the past four decades, only about 60 congenital cases have been documented in the literature ( [10]).

CIFS most frequently arises in distal limbs, while occurrences in the head, neck, trunk, and chest wall are considerably rarer. To date, no cases involving the pleura have been identified in the medical literature ( [11]).

### 5.2 Histological subtypes and behavior

CIFS is classified into two histological subtypes in children: desmoplastic and medullary. The desmoplastic variant is known for its local aggressiveness and histopathological similarity to adult fibrosarcoma. In contrast, the medullary type exhibits less aggressive behavior and generally follows a benign clinical course [12]. Interestingly, the clinical presentation is more influenced by the tumor's location than its subtype.

Tumors involving the chest wall, paravertebral space, or posterior mediastinum often cause pain in the chest, shoulder, neck, or back. When localized to the airways, symptoms such as cough, dyspnea, and hemoptysis are more common. Tumors occupying the pleural cavity, including peripheral intrapulmonary lesions, typically result in pleural effusion. Due to the extreme rarity of CIFS in the thoracic cavity, especially in the pleura, no well-documented symptom profile currently exists for such presentations.

However, a few reported cases of intrathoracic or chest wall CIFS in the literature did present with pneumonia-like symptoms, such as persistent cough, dyspnea, or recurrent respiratory infections unresponsive to antibiotics. This overlap often leads to initial misdiagnosis as pneumonia, similar to our patient's clinical course.

Despite histological similarities to adult-type fibrosarcoma, CIFS constitutes a distinct clinical entity with a more favorable prognosis and unique cytogenetic characteristics. The tumor generally follows a benign course and rarely metastasizes. Metastasis is found in fewer than 10 % of cases in patients under 5 years of age, while over 50 % of patients older than 10 years may develop metastatic disease. Common metastatic sites include the lungs, bones, and occasionally the lymph nodes. Local recurrence occurs in approximately 20–40 % of cases [13].

CIFS is classified as a low-grade myofibroblastic Non-Rhabdomyosarcoma Soft Tissue Sarcoma (NRSTS) ( [14]) Histologically, it is composed of spindle cells arranged in intersecting bundles, often displaying a classic herringbone pattern.

Immunohistochemistry plays a vital role in distinguishing CIFS from other soft tissue tumors with overlapping features, such as infantile fibromatosis and myofibromatosis. CIFS typically shows strong positivity for Vimentin and Smooth Muscle Actin (SMA), with around 25 % of cases positive for Desmin. Markers such as CD34, S-100 protein, and Myogenin are generally negative ( [15]). In our case, the tumor was positive for Vimentin and SMA, focally positive for CD34, and negative for Desmin, S100, and Myogenin. Importantly, molecular testing for the characteristic ETV6–NTRK3 fusion was not available in our center, and this limitation is acknowledged.

Cytogenetic analysis using RT-PCR and FISH can reveal specific genetic markers, including the characteristic t(12;15)(p13;q25) translocation that results in ETV6–NTRK3 gene fusion, a mutation also observed in congenital mesoblastic nephroma. Additionally, CIFS may exhibit polysomies, such as trisomy of chromosomes 11, 8, and 20 ( [16]).

The definitive diagnosis relies on a combination of immunohistochemical findings and the classic histological herringbone architecture, while excluding other entities.

Initial imaging with plain radiography may not clearly reveal the tumor due to nonspecific findings. Ultrasound is useful for prenatal detection and for guiding biopsies of superficial lesions ( [17]).CT scanning is more effective in identifying bony involvement. However, MRI is the preferred imaging modality for assessing tumor size, extent, and its relation to surrounding tissues ( [18]).

Wide Surgical Excision (WSE) is the primary and standard multidisciplinary management approach, especially for chest wall tumors ( [19]).

Achieving complete tumor resection is the most crucial determinant of long-term survival, which can reach up to 90 %. Although neoadjuvant chemotherapy is not universally standardized, it is often administered preoperatively to enhance the chances of total resection. In older children, chemotherapy may also reduce the risk of metastasis.

If microscopic residual disease is present postoperatively, or if full resection is not feasible, adjuvant chemotherapy is typically introduced. The standard regimen includes Vincristine, Cyclophosphamide, and Actinomycin D ( [20]) However, given the young age of most patients, the risk of secondary malignancies from chemotherapy must be carefully weighed ( [21]). Radiotherapy is generally reserved for palliative care due to the potential for developmental complications in pediatric patients ( [22]).

Though pleural involvement in infants is extremely rare, a wide differential must be considered.

The differential diagnosis in our case included infantile fibromatosis, spindle cell rhabdomyosarcoma, synovial sarcoma, and infantile hemangiopericytoma. CIFS was favored due to the presence of a herringbone pattern, strong Vimentin and SMA positivity, focal CD34 reactivity, absence of Desmin, S100, and Myogenin, low Ki-67 proliferative index, and clinical course with no evidence of metastatic spread. These features collectively distinguished it from other spindle cell tumors with overlapping morphology ( [23]).

## 6 Conclusion

Congenital infantile fibrosarcoma involving the pleura is exceedingly rare. Accurate diagnosis requires high clinical suspicion and a multidisciplinary approach. When feasible, surgical resection remains the cornerstone of treatment, often followed by chemotherapy, with prognosis significantly influenced by the completeness of tumor removal.

This case highlights the importance of maintaining a broad differential in infants with pneumonia-like symptoms unresponsive to antibiotics, the necessity of long-term follow-up due to recurrence risk, and the emerging role of molecular testing in guiding access to novel targeted therapies such as NTRK inhibitors.

## 7 Method

The work has been reported in line with the SCARE criteria.

## Abbreviations

B-HCG: β-human chorionic gonadotropin

## Consent of patient

Written informed consent was obtained from the patient's parent/legal guardian for publication and any accompanying images. A copy of the written consent is available for review by the Editor-in-Chief of this journal on request.

## Provenance and peer review

Not commissioned, externally peer-reviewed.

## Ethical approval

Ethical approval is not required at our institution (the Maternity and Children's Hospital-HAMA) by the ethics committee for case reports that do not use patient-identifying information.

## Funding

This research did not receive any specific grant from funding agencies in the public, commercial, or not-for-profit sectors.

## Author contribution

**khaled Alomar:** He was the one who performed the entire surgical procedure and contributed to the diagnosis and follow-up, Conceptualization, resources, who wrote, original drafted, edited, visualized, validated, literature reviewed the manuscript.

**Kamar Shaker:** She contributed to the diagnosis, treatment follow-up, formulation of the comprehensive treatment plan, and periodic follow-up of the case after surgery, resources, who wrote, original drafted, edited, visualized, validated, literature reviewed the manuscript.

**Nidal Alkhani:** Contribution to the diagnosis, therapeutic measures, and follow-up after surgery, edited, visualized, validated, literature reviewed the manuscript.

All authors read and approved the final manuscript.

## Guarantor

Khaled Alomar.

## Research registration

N/A

## Conflict of interest statement

The authors declare that they have no competing interests.

## Data Availability

The datasets generated during and/or analyzed during the current study are not publicly available because the Data were obtained from the hospital computer-based in-house system. Data are available from the corresponding author upon reasonable request.
